# Experiences and Perceived Self-Efficacy in Distance Learning Among Teachers of Students With Special Educational Needs

**DOI:** 10.3389/fpsyg.2021.733865

**Published:** 2021-11-24

**Authors:** Jenny Maurer, Angelika Becker, Johanna Hilkenmeier, Monika Daseking

**Affiliations:** ^1^Educational Psychology, Helmut Schmidt University, University of the Federal Armed Forces Hamburg, Hamburg, Germany; ^2^IDeA Research Center for Individual Development and Adaptive Education of Children at Risk, Frankfurt, Germany; ^3^Faculty of Education, University of Hamburg, Hamburg, Germany

**Keywords:** teachers' self-efficacy, special educational needs, distance learning, COVID-19, learning disorders, DigitLern

## Abstract

The COVID-19 pandemic has had a great impact on school learning so far, creating a new and potentially stressful situation during school closures for teachers and students. The sudden switch to distance learning might have been especially hard to cope with for students with special educational needs (SEN). Teachers of student with SEN might thus face greater obstacles when establishing and dealing with distance learning. Teachers' self-efficacy (TSE) is a well-known factor for students' academic achievement and motivation. Little is yet known about TSE in distance learning, especially not with students with SEN. The present study aimed to investigate the experiences and the perceived TSE in distance learning of teachers teaching students with SEN at special schools and inclusive schools during the COVID-19 pandemic in Germany during June 2020 and January 2021. *N* = 96 teachers from both special schools and inclusive schools were involved in the study and were asked to complete a self-report online questionnaire. The study follows an exploratory design to give a first overview of the experiences of teachers of students with SEN and their TSE during the school closures and distance learning. Results showed that no major difference in overall teaching experiences could be found between teachers teaching at special schools or inclusive schools. The identification of difficulties in reading at distance and the support of students with difficulties in reading at distance was perceived by the teachers as most difficult. Difficulties in writing was being rated significantly less easy to identify at distance than difficulties in mathematics. Further, the support of students with difficulties in mathematics was perceived as being significant more challenging than the identification of difficulties in mathematics. TSE in distance learning was rather low, regardless if the teachers taught at a special school or inclusive school in this time period. TSE correlated positively with the perceived goodness of identification of difficulties and support of students with difficulties in reading, writing, and mathematics. Possible reasons and implications are discussed as well as implications of the overall results for distance learning of students with SEN.

## Introduction

Due to the worldwide COVID-19 pandemic school life and learning changed rapidly in spring 2020 in Germany. In-class learning had to switch very fast to distance learning with little or no in-class schooling. This led to great challenges for both teachers and students. Students with special educational needs (SEN) might be an especially vulnerable group when it comes to difficulties with the adaptation to and the coping with different forms of distance learning. Teachers with students with SEN in their classes might thus have faced more and different challenges in distance learning than teachers without students with SEN in their classes.

There are already a few studies on the experiences of students, their families and teachers during the school closures due to the COVID-19 pandemic from different countries and school systems (e.g., Garbe et al., 2020; Huber and Helm, 2020; König et al., 2020; OECD, 2020; Vuorikari et al., 2020; Steinmayr et al., 2021; Thorell et al., 2021), but only a few studies so far investigated the situation of students with SEN during the COVID-19 pandemic (e.g., Goldan et al., 2020; Nusser, 2021; Scheer and Laubenstein, 2021; Thorell et al., 2021). Those results indicate that students with SEN might go through more negative experiences and more problems whilst dealing with distance learning and challenging situations during school closures than students without SEN. Reich et al. (2020) stated that students with higher school achievement seemed to be less affected by distance learning while more vulnerable student groups were experiencing greater problems with it.

For a better understanding, in the following a short overview of the German school system is given [for more information see the detailed description of the Secretariat of the Standing Conference of the Ministers of Education and Cultural Affairs of the Länder in the Federal Republic of Germany (Kultusministerkonferenz) (Eckhardt, 2019)]. In Germany, all students attend primary school, then after class-level 4 or 6, students attend a secondary school where they either can graduate with basic general education (9th grade, e.g., in German: “Hauptschule”), extensive general education (10th grade, e.g., in German: “Realschule”), or in depth general education (12th or 13th grade, e.g., in German: “Gymnasium”). Further, there are comprehensive schools where different kinds of the degrees mentioned before can be obtained. Beside this, there are special schools (e.g., in German: “Förder-/Sonderschule”), which offer primary education and education to the 10th grade for students with SEN.

Since the United Nations Convention on the Rights of Persons with Disabilities entered into force in Germany in 2009, students with SEN or disabilities shall equally participate in the German educational system, thus an inclusive school system is pursued (Klemm and Preuss-Lausitz, 2017). In an inclusive school system, students with and without SEN are being taught together and learn together (Eckhardt, 2019). If students with SEN are not able to follow the mainstream curriculum, teachers must then prepare different educational plans for their students (Sansour and Bernhard, 2018).

There are eight different so-called support focuses for students with SEN in Germany: learning, emotional and social development, speech, sight, hearing, mental development, physical and motor development and instruction for sick students (Eckhardt, 2019). In 2016, about 7% (*n* = 523.813 students) of all students in compulsory schooling (1st till 9th or 10th grade, depending on school form) had a support focus (Eckhardt, 2019). The three most common support focuses are learning (*n* = 191.169 students, 2.6%), mental development (*n* = 87.516 students, 1.2%), and emotional and social development (*n* = 86.794 students, 1.2%) (Eckhardt, 2019).

At special schools, students are taught by special education teachers. At inclusive schools, the regular teachers are supported by special education teachers, who support students with SEN (Eckhardt, 2019). The decision whether a student with SEN attends a special school or an inclusive school is up to the parents or legal guardians (Eckhardt, 2019).

Various reasons might lead to greater struggles of students with SEN than students without SEN with distance learning. For example, students with SEN often differ from students without SEN regarding their parental socioeconomic status. In Germany, students from families with a low socioeconomic status are three times more often diagnosed with SEN than students from a family with a high socioeconomic status (Kölm et al., 2017). The percentage of students with SEN from families with low socioeconomic status is significant higher at special schools than at inclusive schools (Kölm et al., 2017). A first systematic overview of international studies investigating effects of the COVID-19-related school closures in spring 2020 showed that especially younger students' academic achievement and the academic achievement of students from families with lower socioeconomic status dropped under the school closures (Hammerstein et al., 2021). Helm et al. (2021) came to a similar conclusion in their review on studies that investigated the situation of distance learning during the pandemic-related school closures in Germany, Austria and Switzerland: students from families with low socioeconomic status are disadvantaged in terms of their learning achievement. Among other things, there was a positive association between the socioeconomic status of the families and students' learning success, students' learning motivation, parental competencies to support students in learning as well as the technical equipment of families during distance learning observed (Helm et al., 2021). Conversely, students from families with low socioeconomic status showed lower learning success, lower motivation to learn, less parental competencies for support in their learning, and had reportedly less technical equipment during distance learning.

At the beginning of the COVID-19 pandemic, teachers and schools in Germany were partially not well-prepared for distance learning, especially concerning digital learning (Runge et al., 2021). It should be noted that distance learning does not necessarily involve digital learning, but all different forms of learning that are not carried out in-class. Regarding digital learning, the International Computer and Information Literacy Study (ICILS) showed that before the COVID-19 pandemic, not even one-third of teachers in Germany had received further training in digital learning and teaching (Eickelmann et al., 2019). With regard to the use of digital media by students with SEN, further training of teachers was as low as 4.6% (Eickelmann et al., 2019). A study from Huber and Helm (2020) carried out in Germany, Austria, and Switzerland, in which also students, parents, and teachers were surveyed during school closures in 2020 revealed that Germany's education system lags behind in terms of several aspects regarding digital learning and teaching and not only teachers but also schools were not well-prepared for distance learning. Technical capacities and resources for digital learning were significantly rated lower by the school staff (most of them teachers) in Germany than in its neighboring countries Austria and Switzerland (Huber and Helm, 2020). Therefore, 56% of the school staff disagreed or strongly disagreed with the statement that “technical capacities in the school are sufficient for web-based formats” (Huber and Helm, 2020, p. 251). Likewise, the school staff in Germany rated their digital competencies significantly lower than school staff in Austria or Switzerland (Huber and Helm, 2020). Furthermore, digital competencies of the school staff were associated with technical resources of the schools for digital learning (Huber and Helm, 2020). During the school closures, the majority of students in Germany spent most of their time doing school assignments in self-study, with little contact to their teachers (Thorell et al., 2021). Teachers' feedback to students, as well as teachers' individual support, again seems to work better in Germany's neighboring countries (Huber and Helm, 2020). It was shown that technical capacities of schools were positively associated with more feedback and individual support for students from teachers (Huber and Helm, 2020). Altogether, the situation in schools in Germany during the school closures 2020 was stressful for a large proportion of both teachers and students (Huber and Helm, 2020).

Some studies also investigated specifically the situation of students with SEN during school closures in Germany. Nusser (2021) examined in a study differences between students with SEN and without SEN. During the school closures 2020, students with SEN spent more than twice as many hours studying as students without SEN (16 vs. 35 hours per week) (Nusser, 2021). Likewise, these students also were supported by their parents more than twice as many hours with their schoolwork than students without SEN (5 vs. 11 hours per week) (Nusser, 2021). Another study investigating experiences during school closures in 2020 in several European Countries observed that a large proportion of parents of German students with SEN reported that whilst special educational support was given to them (more than 70%), the amount of given support was not sufficient. Likewise, two thirds of parents reported that no communication with the school about the special educational support had taken place (Thorell et al., 2021). A study by Scheer and Laubenstein (2021) also shows that students with support focus in emotional and social development could not adapt as well to distance learning as students without SEN. Similarly, they were more likely to be exposed to psychosocial hazards than students without SEN (Scheer and Laubenstein, 2021). Further, externalizing problems increased slightly in these students during distance learning (Scheer and Laubenstein, 2021). Moreover, a support focus in emotional and social development is associated with a decrease in emotional well-being related to school during distance learning (Scheer and Laubenstein, 2021). Based on investigations of an experimental school for inclusive education, the following aspects were summarized to support students with SEN well, even in distance learning: adapted individual tasks, sufficient feedback by teachers, a good relationship and contact between teachers and students as well as their parents (Goldan et al., 2020). Becker et al. (2020) indicated that students with ADHD showed more difficulties with distance learning than their peers without an ADHD diagnosis that are not only due to preexisting academic problems. The authors stress that schools need to provide support especially to students with mental health and/or learning difficulties. There is a big intersection between students with ADHD and SEN (see for example representative data of the US: Schnoes et al., 2006). Thus, investigating the effects of school closures on students with SEN has been identified as a key research priority (Holmes et al., 2020).

It is important to point out that in Germany the average level of achievement between students with SEN and students without SEN differs. Students with SEN show a mean delay in school achievement of at least 2 years compared to students without SEN (Wocken and Gröhlich, 2007). Students with SEN in special schools have significantly lower skills in reading, mathematics, and science than students without SEN in inclusive schools (Müller et al., 2017). Thus, if students with SEN already had a delay in school achievement before the COVID-19 pandemic this gap could now even widen more.

The above described problems in digitalization at German schools, uncertainties and new challenges in distance learning might have led to a low teachers' self-efficacy (TSE) during the school closures in spring 2020. TSE is a well-known and studied factor for successful teaching and instructional practice as well as for students' academic achievement and motivation as well for the emotional well-being of teachers (e.g., Klassen et al., 2009; Zee and Koomen, 2016). Thus, TSE might be a major factor in the successful implementation and establishment of distance learning.

In general, self-efficacy is understood as the conviction that an effect can be achieved through one's own actions (Bandura, 1977, 1997). In accordance to Bandura (1977, 1997); Skaalvik and Skaalvik (2010) define TSE as “individual teachers' beliefs in their own ability to plan, organize, and carry out activities that are required to attain given educational goals.” (p. 1059).

A few studies have already looked at TSE in school closures during the COVID-19 pandemic (e.g., König et al., 2020; Börnert-Ringleb et al., 2021; Kast et al., 2021). Börnert-Ringleb et al. (2021) reported that during the COVID-19 pandemic TSE related to the use of digital learning in special needs education (special and inclusive schools) in Germany is a predictor for the perceived quality of digital learning, whereas a more generalized TSE is not a predictor. In another study conducted in Austria, TSE during the school closures was significantly lower with regard to students with SEN compared to a group of students with high academic achievements in school and a control group (Kast et al., 2021). König et al. (2020) showed that TSE is a predictor for successful adapting tasks to the students' demands and giving feedback to students during school closures.

Before the COVID-19 pandemic, Viel-Ruma et al. (2010) observed in a study no significant differences between TSE of special needs educators in different teaching settings (self-contained, resource, or inclusion). In inclusive schooling, Schwab (2019) found that the TSE of special education teachers were higher than of regular teachers toward students with SEN. Furthermore, it can be assumed that teachers with more years of experience in teaching have a higher TSE than teachers with less years of experience in teaching (Flores et al., 2004).

Given that students with SEN might especially struggle with distance learning and that the TSE in regard to distance learning might be one of the crucial factors for successful distance learning, this study is focusing among other experiences at the TSE of teachers teaching students with SEN.

In this study, we followed an exploratory approach and tried to get a broad descriptive overview to get a first insight of the overall situation of teachers of students with SEN during the first year of the COVID-19 pandemic. We addressed following questions: Did the number of hours per week teachers used digital learning before and since the COVID-19 pandemic change? How could teachers identify difficulties and support students with difficulties in reading, writing, and mathematics in the context of distance learning? How was the perceived TSE in distance learning in supporting students with SEN? Is there an association between TSE in distance learning and the identification of difficulties as well as the support of students with difficulties in reading, writing or mathematics in distance learning? Are there factors within the teachers that might have had an influence on the perceived TSE (e.g., gender, age, or years of work experience)? Furthermore, for all of these questions, we investigated whether there are differences between teachers at special schools and inclusive schools.

## Materials and Methods

### Procedure and Instruments

This study was conducted between June 2020 and January 2021 within the project DigitLern with an anonymous self-report online questionnaire. Teachers throughout Germany were invited to participate in the study via e-mail by distribution lists of special education associations. Before participation informed consent was given.

A self-developed questionnaire was used that included demographic data of the teachers [gender (male, female, diverse), age, years of work experience], data related to their teaching experiences (school form where the teachers work, number of students with special educational needs, support focuses of those students), and data related to distance learning (used devices in distance learning, perceived helpful methods in distance learning, hours of digital learning used in teaching). Further, possibilities of identification of difficulties and support of students with difficulties in reading, writing, and mathematics at distance, as well as the TSE in distance learning were ascertained.

For the factors school form [e.g., primary school, school where students can graduate with basic general education (e.g., Hauptschule), special school] and support focuses (e.g., support focus in learning, support focus in emotional and social development), multiple answers were possible. There were predefined answers for helpful methods (e.g., learning apps, worksheets; 0 = not helpful to 4 = helpful) and used devices (e.g., computer, laptop; 0 = never to 4 = always). Goodness of identification of difficulties as well as the support of students with difficulties in reading, writing, and mathematics at distance were assessed with single items (e.g., “Difficulties in reading can be well identified at distance.”; “Students with difficulties in reading can be supported well at distance”; 0 = disagree; 1 = rather disagree; 2 = undecided 3 = rather agree; 4 = agree). As the identification of difficulties and support of students with difficulties in the academic skills reading, writing, and mathematics requires domain-specific material and competencies (e.g., Ise et al., 2012a,b), teachers were asked to answer separately for all three disciplines.

TSE in distance learning was assessed by a self-developed scale. Although there exist quite a couple of TSE scales in general, no existing and already evaluated and validated TSE scale that posed questions fitting to the research question here was found. Therefore, the authors decided to go with an exploratory approach and developed the scale by themselves. The scale contains twelve items (e.g., “I experience teaching at distance as effective.”) with a five-point response scale (0 = disagree; 1 = rather disagree; 2 = undecided 3 = rather agree; 4 = agree).

For the development of the TSE scale, a principal component analysis (PCA) with orthogonal rotation was performed with the 13 items. The Kaiser-Meyer-Olkin (KMO) value, which indicates the sampling adequacy, was 0.886 for all 13 variables. A value >0.8 is meritorious (Kaiser and Rice, 1974). Bartlett's test was significant. Because of the high KMO value and significant Bartlett's test, a PCA could be performed.

The PCA showed that two components had an eigenvalue above 1.0 and an explained variance above 10%. The first component had an eigenvalue of 6.58 and an explained variance of 50.6%. The second component showed an eigenvalue of 1.33 and an explained variance of 10.2%. Further, the scree plot indicated one component. Due to the low eigenvalue and the low explained variance of the second component as well as the scree plot's indication, a one-component solution was chosen.

The analysis was performed again with one fixed component. Thus, one variable showed a loading below 0.3. This variable was excluded from the scale. The TSE scale was finally formed with 12 items. The final scale shows high internal consistency (*Cronbach's* α = 0.92; *n* = 90).

### Participants

In total *N* = 118 teachers answered the questionnaire. For the data cleansing, all participants who answered the questionnaire in a time span too short to be able to answer the questionnaire reasonable (<5 minutes) were excluded (*n* = 11). Further, teachers who do not teach any students with special educational needs were excluded from the data (*n* = 11). The final sample included *N* = 96 teachers (female = 81 (84.4%); age: *M* = 46.48, *SD* = 11.20; years of work experience: *M* = 18.38, *SD* = 10.83).

More than half of them (*n* = 52; 54.2%) taught in special schools. For group comparisons, two groups of teachers were formed. The first group includes teachers from special schools, the second groups includes teachers, who work at inclusive schools. Five of the 52 teachers reported working at a special school as well as at an inclusive school. For the analysis, they were placed in the group of special school teachers. A chi-^2^ test and unpaired *t*-test were performed to determine if both groups of teachers (teachers at special schools and teachers at inclusive schools) are comparable to each other regarding gender, age, and years of work experience.

### Analysis

First, the data was analyzed descriptively. For the scale of TSE in distance learning means were calculated. For this, a maximum of 30% missing values was tolerated. For one person no mean could be calculated.

*T*-tests (unpaired and paired) were conducted to determine differences between teachers at special schools and inclusive schools regarding hours of digital learning and further differences in digital learning before the COVID-19 pandemic and since the COVID-19 pandemic.

To analyze if there were differences between the perceived goodness of how the teachers could respond to the students' needs in distance learning in regard to the different academic skills, the identification of difficulties and support of students with difficulties in those academic skills and between the school forms where the teachers taught, a mixed analyses of variance (ANOVA) was conducted. It included the within factor academic skills (reading vs. writing vs. mathematics), the within factor handling (identification of difficulties vs. support of students with difficulties) and the between factor school form taught at (special school vs. inclusive school). To test the assumptions of sphericity, mauchly's test was conducted for the main and interaction effects. The assumption of sphericity was violated for the main effects of academic skills, χ^2^(2) = 37.405, *p* < 0.001. For the corrections of the degrees of freedom, Greenhouse-Geisser test was used (ε = 0.74). As effect size, partial eta-square was calculated. According to Cohen (1988), for the partial eta-square there is a small effect at $\eta_{part}^{2}$ = 0.01, a medium effect at $\eta_{part}^{2}$ = 0.06, and a large effect at $\eta_{part}^{2}$ = 0.14. Then *t*-tests were conducted to further explore possible differences between the perceived goodness of identification of difficulties and the support of students with difficulties and the different academic skills. In the *t*-tests, the between factor school taught at was not taken into account, since the ANOVA did not reveal any differences between the two groups of teachers.

To further explore possible differences in the TSE in distance learning between teachers of special schools and inclusive schools, unpaired *t*-tests were computed.

Furthermore, correlation analyses (Pearson correlations) were performed to detect associations between gender, age, and years of work experience with the TSE at distance learning. Also, correlations analyses (Pearson correlations) were conducted to explore associations between TSE in distance learning with the identification of difficulties as well as the support of students with difficulties in reading, writing, or mathematics in distance learning. Effect sizes according to Cohen (1988) are small at *r* = 0.10, medium at *r* = 0.30 and large at *r* = 0.50. Since the previous *t*-test did not identify any difference between the two groups of teachers (teachers at special schools or inclusive schools) in terms of TSE, the correlation analyses were performed for all teachers.

The size of the *n* varies among the analyses. In order not to reduce the sample size further, no listwise exclusions were made here. A significance level of α = 0.05 was set for all analyses. The IBM SPSS Statistics Version 27 program from IBM Corp. was used for data analysis.

## Results

### Descriptive Analyses

The two subsamples (special school teachers and inclusive school teachers) are comparable to each other and do not differ significantly regarding gender, age, and work experience (see Table 1).

**Table 1:** Gender, age, and years of work experience.

		**Gender**		**Age**		**Years of work experience**	
	***n***	**female *n* (%)**	**male *n* (%)**	***M***	***SD***	***M***	***SD***
Teachers at special schools	52	43 (82.7)	9 (17.3)	45.88	11.32	17.86	10.68
Teachers at inclusive schools	44	38 (86.4)	6 (13.6)	47.18	11.14	19.00	11.10
All teachers	96	81 (84.4)	15 (15.6)	46.48	11.20	18.38	10.83

*All teachers = teachers of special schools and teacher of inclusive schools. No significant differences between teachers of special schools and teachers of inclusive schools*.

More than half of the teachers from inclusive schools work at primary schools (*n* = 22; 50%). For further detail, see Table 2.

**Table 2:** School forms of the inclusive schools teachers work at.

	***n***	***%***
Primary school	22	50.0
School where students can graduate with basic general education (e.g., “Hauptschule”)	6	13.6
School where students can graduate with extensive general education (e.g., “Realschule”)	4	9.1
School where students can graduate with in-depth general education (e.g., “Gymnasium”)	1	2.3
Comprehensive forms (e.g., “Gesamtschule”)	13	29.5
Other	5	11.4

*Multiple answers possible*.

Teachers at inclusive schools teach an average of 12.63 students with SEN (see Table 3). In both school forms the range for taught students per teacher varies widely.

**Table 3:** Number of students with SEN.

	***n***	***M***	***SD***	**min**.	**max**.
Teachers at special schools	52	20.38	17.36	2	96
Teachers at inclusive schools	43	12.63	12.21	2	78

At special schools and inclusive schools the largest groups of students have the support focus in learning (special schools: *n* = 31; 59.6%; inclusive schools: *n* = 40; 90.9%) as well as in emotional and social development (special schools: *n* = 24; 46.3%; inclusive schools: *n* = 33; 75%), (see Table 4).

**Table 4:** Support focuses.

	**Teachers at special schools**		**Teachers at inclusive schools**		**All teachers**	
**Support focus**	***n***	**%**	***n***	**%**	***n***	**%**
Learning	31	59.6	40	90.9	71	74.0
Emotional and social development	24	46.2	33	75.0	57	59.4
Mental development	20	38.5	18	40.9	38	39.6
Physical and motor development	10	19.2	19	43.2	29	30.2
Other	14	26.9	18	40.9	32	33.3

*Answered by teachers; multiple answers possible; N = 96; teachers at special schools n = 52; teachers at inclusive school n = 44; all teachers = teachers of special schools and teachers of inclusive schools*.

The two devices that were most frequently identified as often or always used for distance learning by teachers from special schools were laptop (*n* = 29; 55.8%) and smartphone (*n* = 26; 50%). Teachers from inclusive schools identified most frequently laptop (*n* = 29; 65.9%) and telephone (*n* = 24; 54.6%) as often or always used for distance learning.

The methods that most teachers at special schools rated as helpful or somewhat helpful were worksheets (*n* = 38; 73.1%), visual aids (*n* = 36; 69.2%), and working with exercise books and/or textbooks (*n* = 33; 63.5%). The methods that most teachers at inclusive schools rated as helpful or somewhat helpful were also worksheets (*n* = 39; 88.6%) and working with exercise books and/or textbooks (*n* = 30; 68.2%), as well as visual aids (*n* = 29; 65.9%) and learning apps (*n* = 29; 65.9%).

### Digital Learning

The analyses neither showed significant differences in hours of digital learning used in teaching before the COVID-19 pandemic between teachers of special schools and teachers of inclusive schools [*t*(88) = −0.938; *p* = 0.351], nor during the survey period [*t*(86) = −0.049; *p* = 0.961].

Significant differences were found between hours of digital learning before the COVID-19 pandemic and during the survey period (see Table 5). The average number of hours has more than doubled. These results were evident for both subsamples.

**Table 5:** Differences between hours of digital learning before the COVID-19 pandemic and since the COVID-19 pandemic regarding school form.

	**Hours per week before the COVID-19 pandemic**					**Hours per week since the COVID-19 pandemic**					***t*** **-test**		
	***n***	***M***	***SD***	**min**.	**max**.	***n***	***M***	***SD***	**min**.	**max**.	***t***	***df***	***p* (2-sited)**
Special schools	48	2.38	4.09	0	21	48	8.23	9.08	0	36	−4.413	47	<0.001
Inclusive schools	40	3.40	5.83	0	25	40	8.33	9.12	0	40	−2.901	39	0.006

*Answered by teachers*.

Furthermore, a wide range before the COVID-19 pandemic in the number of hours of digital learning was observed. The range was even wider since the school closures related to the beginning of the COVID-19 pandemic.

### Identification of Difficulties and Support of Students With Difficulties in Reading, Writing, and Mathematics

All means of stated goodness of identification of difficulties and support of students with difficulties in reading, writing, and mathematics are rather low (see Tables 6, 7).

**Table 6:** Differences between reading, writing, and mathematics regarding identification and support.

		**Reading**		**Writing**		**Mathematics**		***t*** **-test**		
	***n***	***M***	***SD***	***M***	***SD***	***M***	***SD***	***t***	***df***	***p* (2-sited)**
Identification	94	1.10	1.10	1.45	1.21	–	–	−2.703	93	0.008
	93	1.10	1.08	–	–	1.66	1.156	−4.591	92	<0.001
	92	–	–	1.42	1.21	1.64	1.154	−2.418	91	0.018
Support	93	0.97	0.85	1.27	0.99	–	–	−3.493	92	0.001
	94	0.97	0.85	–	–	1.28	1.031	−3.184	93	0.002
	93	–		1.27	0.99	1.28	1.036	−0.179	92	0.859

*Answered by teachers. Answers could be given on a five-point response scale (0 = disagree; 1 = rather disagree; 2 = undecided; 3 = rather agree; 4 = agree)*.

**Table 7:** Differences between identification of difficulties and support of students with difficulties in reading, writing, and mathematics.

		**Identification**		**Support**		***t*** **-test**		
	***n***	***M***	***SD***	***M***	***SD***	***t***	***df***	***p* (2-sited)**
Reading	94	1.12	1.10	0.97	0.85	1.620	93	0.109
Writing	92	1.45	1.22	1.26	0.99	1.571	91	0.120
Mathematics	93	1.66	1.16	1.28	1.04	4.166	92	<0.000

*Answered by teachers. Answers could be given on a five-point response scale (0 = disagree; 1 = rather disagree; 2 = undecided 3 = rather agree; 4 = agree)*.

Significant main effects were found for the factor academic skills, *F*(1.49, 132.22) = 12.726, *p* < 0.001, $\eta_{part}^{2}$ = 0.13 as well as for the factor handling, *F*(1, 89) = 8.157, *p* = 0.005, $\eta_{part}^{2}$ = 0.08. The interaction of the within factor academic skills and the factor handling was significant, *F*(2, 178) = 3.396, *p* = 0.036, $\eta_{part}^{2}$ = 0.04. None of the interactions with the between factor school form reached significance, pointing to no differences between teachers teaching at special schools or at inclusive schools.

*T*-tests (see Table 6) showed that teachers perceived it harder to identify difficulties in reading at distance than difficulties in writing and mathematics. Teachers' rated also that difficulties in writing are significantly less easy to identify at distance than difficulties in mathematics. Teachers rated the support of students with difficulties in reading as significantly harder at distance than the support of students with difficulties in writing and mathematics at distance.

Further, a significant difference between the perceived goodness of identification of difficulties in mathematics and support of students with difficulties in mathematics at distance could be found. For difficulties in reading and writing, no significant differences were found.

### Teachers' Self-Efficacy in Distance Learning

Analyses show that TSE in distance learning is generally low for all teachers (see Table 8). No significant differences between teachers of special schools and teacher of inclusive schools could be found [*t*(93) = −0.204; *p* = 0.838].

**Table 8:** Teachers report of their TSE in distance learning.

	***n***	***M***	***SD***	**min**.	**max**.
Teachers at special schools	52	1.18	0.79	0	2.92
Teachers at inclusive schools	43	1.21	0.71	0.08	2.75
All teachers	95	1.19	0.75	0	2.92

*Answers could be given on a five-point response scale (0 = disagree; 1 = rather disagree; 2 = undecided; 3 = rather agree; 4 = agree). All teachers = teachers of special schools and teachers of inclusive schools*.

TSE in distance learning is not associated with gender, age nor years of work experience (see Table 9). However, an association could be found between TSE in distance learning and perceived goodness of identification of difficulties as well as support of students with difficulties in reading, writing, and mathematics (see Table 10).

**Table 9:** Correlations between teachers' gender, age, years of work experience, and TSE in distance learning.

		**TSE**		
		***n***	***r***	***p* (2-sited)**
All teachers	Gender	95	0.00	0.978
All teachers	Age	95	0.08	0.434
All teachers	Years of work experience	95	−0.04	0.726

*Point-biseral and Pearson-correlation. All teachers = teachers from special schools and teachers from inclusive schools. No separate analyses for teachers of special schools and teachers of inclusive schools because they are not significantly different in terms of age and work experience nor in their TSE in distance learning*.

**Table 10:** Correlations between identification of difficulties as well as support of students with difficulties in reading, writing, and mathematics and TSE in distance learning.

			**TSE**		
			***n***	***r***	***p* (2-sited)**
All teachers	Identification	Reading	94	0.36	<0.001
		Writing	93	0.51	<0.001
		Mathematics	93	0.53	<0.001
	Support	Reading	94	0.51	<0.001
		Writing	93	0.72	<0.001
		Mathematics	94	0.68	<0.001

*Pearson-correlation. All teachers = teachers from special schools and teachers from inclusive schools. No separate analyses for teachers of special schools and teachers of inclusive schools because they are not significantly different in their TSE in distance learning*.

## Discussion

This study provides important results, which give a first impression on the experiences with distance learning of teachers teaching students with SEN at special schools and at inclusive schools in Germany during the COVID-19 pandemic. No significant differences between teachers of special schools and teachers of inclusive schools regarding the use of digital learning, in the perceived goodness of identification of difficulties and support of students with difficulties in reading, writing, and mathematics, as well as the TSE in distance learning in general was observed here.

The results for all teachers surveyed show that teachers perceived difficulties in reading to be significantly less easy to identify than difficulties in writing and mathematics at distance. Teachers also perceived the support of students with difficulties in reading as less easy than those with difficulties in writing or in mathematics at distance. Likewise, teachers rated students with difficulties in writing significantly less easy to be identified at distance than students with difficulties in mathematics. Further, the support of students with difficulties in mathematics is perceived significantly more difficult than the identification of difficulties.

The TSE in distance learning stated by the teachers is rather low. In addition, positive correlations between identification of difficulties as well as support of students with difficulties in reading, writing, and mathematics and TSE in distance learning could be found.

### Teachers at Special School and Teachers at Inclusive Schools

The two groups of teachers were comparable in regard to gender, age, and years of work experience. In the descriptive analyses, the three most frequent support focuses of students supported by the teachers of special schools and teachers of inclusive schools are the support focuses in learning, emotional and social development as well as mental development. This is consistent with the most common support focuses in Germany (Eckhardt, 2019). Further, no major differences between the two groups of teachers with regard to the used devices and the most helpful methods could be investigated. As the most helpful methods in distance learning were rated no digital methods, but paper and books. Another study from Germany also shows that, especially in elementary school, tasks in distance learning are set with paper (Dincher and Wagner, 2021). This shows that distance learning in this sample of teachers of students with SEN is by definition not digital learning, but digital learning is one part of distance learning.

### Digital Learning

For digital learning before the COVID-19 pandemic and digital learning since the COVID-19 pandemic no significant differences between both teacher groups could be obtained. However, significant differences were determined for all teachers in hours of digital learning before the COVID-19 pandemic and since the COVID-19 pandemic with a significant larger amount of digital learning after the beginning of the school closures due to the COVID-19 pandemic. Our data shows that a large part of distance learning is still conducted offline with worksheets, paper, and books. In addition, in the observed period, schools were not closed the whole time entirely, but many different approaches in schooling due to the pandemic containment were seen in Germany (e.g., alternating presence teaching or hybrid lessons).

### Identification of Difficulties and Support of Students With Difficulties in Reading, Writing, and Mathematics

Moreover, low values could be determined for both teacher groups regarding the perceived identification of difficulties and support of students with difficulties in reading, writing, and mathematics. Apparently, difficulties in reading are perceived to be most difficult to identify and students with difficulties in reading are perceived to be the most difficult to support at distance, compared to writing, and mathematics. Likewise, difficulties in mathematics were perceived to be identified easier than difficulties in writing at distance. These results may be due to the fact that identification and support in different skills (reading, writing, and mathematics) require different competencies and materials. Previous research showed that precise identification and support of specific skills or competencies – in contrast to general supporting strategies – is crucial for a positive development of domain-specific competencies in the acquisition of academic skills (Ise et al., 2012a,b). Further research in this area is needed to explore the reasons for the differences between the academic skills (reading, writing, and mathematics) further.

The result that supporting students with difficulties in mathematics at distance is perceived significantly more challenging than identifying difficulties in mathematics at distance is supported by another study in which educational therapists were surveyed and who were asked identical questions about identification of difficulties and support of students with difficulties in reading, writing, and mathematics (Maurer et al., 2021). Based on a systematic review, Lafay et al. (2019) suggested that students with difficulties in mathematics could potentially benefit from using concrete or virtual materials (so-called manipulatives e.g., blocks or play money) in learning mathematics.

It might be possible that there are difficulties in supporting students with difficulties in mathematics in distance learning due to a lack of use of concrete materials. However, more research is needed to explore this topic further.

However, these results are very important for the time after the COVID-19 pandemic and thus, after the distance learning. It could be suggested due to the challenges in the identification of difficulties and support of students with difficulties in reading, writing, and mathematics, there is a great need for support in the matter of these academic skills in students with SEN.

### Teachers' Self-Efficacy in Distance Learning

Furthermore, this study shows that TSE in distance learning is generally low for all teachers of students with SEN, regardless of whether teachers taught at special or at inclusive schools. Because this was an online survey, it can be assumed that teachers with a greater affinity for digital media are more likely to have participated. Therefore, it could be assumed that the TSE in distance learning is possibly even lower for teachers with less affinity for digital media. Further, no significant differences could be found between both teacher groups. Studies conducted before the COVID-19 pandemic point also to no significant differences regarding TSE of teachers in special education between different teaching settings (Viel-Ruma et al., 2010). The low TSE for both, teachers from special schools and inclusive schools in this study could be due to the fact that the teachers surveyed teach students with SEN. A previous study would support these assumption, which already found that TSE during school closures was significantly lower in regard to students with SEN than to students with high achievement and a control group (Kast et al., 2021). Börnert-Ringleb et al. (2021) stated in their study that during the COVID-19 pandemic the TSE regarding the use of digital learning in special needs education is a predictor for the perceived use of digital learning. This fits with the results of the present study, because the TSE is low in this sample and the methods identified by teachers as most helpful were not digital methods, but based on paper and books. After all, two thirds of teachers of inclusive schools still named learning apps as helpful or somewhat helpful. In this study, the TSE in distance learning is not related to gender, age, nor years of work experience. Hence, the results are not consistent with previous findings with respect to TSE and years of work experience (Flores et al., 2004). Perhaps this is due to the fact that teachers have not had any experience with distance learning in their careers so far and the situation during the COVID-19 pandemic is new and challenging for everyone, regardless of gender, age, and work experience. However, a positive correlation between TSE and the perceived identification of difficulties as well as the support of students with difficulties could be found. On the one hand, this means that teachers with a higher TSE are more likely to identify difficulties in reading, writing, and mathematics. And that they feel they can better support students with difficulties in reading, writing, and mathematics at distance.

Promoting TSE in distance learning of teachers teaching students with SEN is essential. In the case of possible further school closures and distance learning in the future, teachers teaching students with SEN should be better prepared for distance learning (e.g., training in the use of digital learning in regard to students with SEN, intervisions regarding e.g., methods between the teachers), which would probably increase TSE in distance learning of teachers of students with SEN. This could promote positive effects like students' school achievement and their motivation to study (Zee and Koomen, 2016). This would be especially important for students with SEN, as these students are likely to be particularly disadvantaged and are more struggling by the distance learning during the COVID-19 pandemic (Reich et al., 2020; Scheer and Laubenstein, 2021).

## Limitations

As an ad-hoc study, the results only provide insight into the distance learning situation of teachers of students with SEN during one time period of the COVID-19 pandemic in Germany. The fact that the survey was conducted online may have resulted in a sample selection. Therefore, the results cannot be generalized.

In this study the focus was laid on the teachers' experiences and perceptions only. Conclusions were only made by a self-reported questionnaire. Further studies should explore the perspective of students with SEN further as well as the effects of the school closures on academic achievements and learning motivations of students with SEN.

Moreover, due to the sample size, no distinction was made between the different support focuses. Maybe there will be found differences in future studies.

Also, comparisons between students with SEN and students without SEN should be considered. There is a risk, that due to the school closures students with SEN might fall behind students without SEN further regarding school achievement and motivation. Which could widen the gap of achievement levels between these group of students.

## Conclusion

Due to the special learning situations that have arisen as a result of the COVID-19 pandemic, special attention must be paid to students with SEN (Holmes et al., 2020). This study is a further contribution to bringing the needs of these students and their supporting teachers into focus.

The results of this study are essential to students with SEN and their teachers. It is important to identify challenges in distance learning for students with SEN early to enable them for an equal opportunity for learning and participation.

To increase TSE in teachers of students with SEN digital skills and equipment should be promoted.

Another focus should be on identifying difficulties and supporting students with difficulties in reading, writing, and mathematics, on the one hand in case schooling have to be held at distance again and on the other hand to compensate and reduce any deficits that may have arisen during the distance learning.

## Data Availability Statement

The datasets presented in this article are not readily available because all research questions have not yet been addressed. Requests to access the datasets should be directed to Jenny Maurer, j.maurer@hsu-hh.de.

## Ethics Statement

Ethical review and approval was not required for the study on human participants in accordance with the local legislation and institutional requirements. The participants provided their written informed consent to participate in this study.

## Author Contributions

JM, AB, JH, and MD contributed to conception, design of the study, and wrote sections of the manuscript. JM organized the database, performed the statistical analysis, and wrote the first draft of the manuscript. All authors contributed to manuscript revision, read, and approved the submitted version.

## Funding

The open access publication fees were funded by the library of the Helmut Schmidt University/University of the Federal Armed Forces Hamburg.

## Conflict of Interest

The authors declare that the research was conducted in the absence of any commercial or financial relationships that could be construed as a potential conflict of interest.

## Publisher's Note

All claims expressed in this article are solely those of the authors and do not necessarily represent those of their affiliated organizations, or those of the publisher, the editors and the reviewers. Any product that may be evaluated in this article, or claim that may be made by its manufacturer, is not guaranteed or endorsed by the publisher.
